# Effect of *Bacillus megaterium*-Coated Diets on the Growth, Digestive Enzyme Activity, and Intestinal Microbial Diversity of Songpu Mirror Carp *Cyprinus specularis* Songpu

**DOI:** 10.1155/2020/8863737

**Published:** 2020-11-13

**Authors:** Liang Luo, Qiyou Xu, Wei Xu, Jinnan Li, Chang'an Wang, Liansheng Wang, Zhigang Zhao

**Affiliations:** Heilongjiang River Fisheries Research Institute, Chinese Academy of Fishery Sciences, Harbin, Heilongjiang 150070, China

## Abstract

The present study was conducted to evaluate the effect of a *Bacillus megaterium*-coated diet on growth performance, digestive enzymes, and intestinal microbial diversity in Songpu mirror carp (*Cyprinus specularis* Songpu). The fish were manually fed two diets (a control diet and a *B. megaterium*-coated diet) three times daily until apparent satiation for 56 days. Compared with the control group, supplementation with the *B. megaterium*-coated diet enhanced the fish growth and significantly reduced the feed conversion ratio (*P* < 0.05). The activities of foregut amylase and lipase in the treatment group were significantly higher than those in the control group (*P* < 0.05). The activities of foregut, midgut, and hindgut proteases in the treatment group were all higher than those in the control group (*P* > 0.05). The results of sequencing the 16S rDNA genes of the microbiota through high-throughput sequencing showed that the diversity and abundance of the intestinal microflora increased along with Songpu mirror carp growth. The Songpu mirror carp fed a diet coated with *B. megaterium* displayed increased proportions of intestinal *Bacillus* and *Lactococcus* at the genus level, and both were significantly higher than those of the control group (*P* < 0.05). These results therefore suggest that dietary *B. megaterium* application can improve the growth and digestive enzyme activity of Songpu mirror carp and enrich the beneficial genus composition of its main intestinal microflora.

## 1. Introduction

Fish, as one of the main dietary sources of animal protein, play a very important role in the human food structure. However, in recent years, aquatic product safety has become one of the biggest factors hindering the development of aquatic products due to concerns about the safety of hormones, antibiotics, and excessive microorganisms [1]. With the rapid development of high-density intensive farming, the aquaculture water environment is deteriorating daily, resulting in disease outbreaks and even mass deaths of farmed animals. There is an urgent need to find environmentally friendly and safe alternatives to ensure the healthy growth of farmed animals [2].

As a new feed additive after the era of antibiotics, probiotics are considered to be important substitutes for feed antibiotics [3–5]. Studies have shown that the use of probiotics not only can promote the growth of aquatic animals and improve their survival rate but also can reduce the incidence of aquatic animal diseases [6, 7]. *Bacillus* is a saprophytic gram-positive bacterium commonly found in the breeding environment [8]. The research and application of *Bacilli* in the field of aquaculture have attracted much attention. The first application of probiotics in aquaculture was carried out by balancing the bacterial population in the water, and it achieved good results [9]. Some studies have shown that adding *Bacillus subtilis* to feed not only can promote the growth of aquatic animals and improve the activities of their digestive enzymes and their nonspecific immunity but also can improve the structure of their intestinal microflora [10–12]. In the middle of the 20th century, Chinese scholars began to study *Bacillus megaterium*, mainly focusing on screening of strains, particularly their ability to break down organic matter, their nitrogen metabolism, and their application as probiotics in aquaculture wastewater treatment [13–17].

The common carp (*Cyprinus carpio*) is the most extensively cultured freshwater fish species in China, and the production of common carp was 2,962,218 t in 2018 [18]. Songpu mirror carp (*Cyprinus specularis* Songpu), a variety of common carp, accounts for an increasingly larger proportion of production due to its relatively faster growth, better disease resistance, higher meat conversion rate, and near absence of scales on the body surface [19, 20]. However, with the expansion and promotion of high-density farming, increasing feeding frequency and water pollution, the problems of food safety and quality have become increasingly serious.

Nutrients are among the most important and easily regulated factors affecting the resistance of aquatic animals. Food microbiology includes microorganisms that have both beneficial and deleterious effects on food quality and safety and may therefore be of concern for the public health. In this study, we aimed to evaluate the effect of *B. megaterium*-coated diets on the growth, digestive enzyme activity, and intestinal microbial diversity in Songpu mirror carp and to provide a theoretical basis for the practice of healthy and ecological fish farming.

## 2. Materials and Methods

### 2.1. Diet Preparation

*B. megaterium* was prepared in our laboratory [17]. The content of *B. megaterium* was 1 × 10^9^CFU/ml. The commercial feed was purchased from Zhejiang AIPHA Feed Co., Ltd., China. The guaranteed values of the feed product composition analysis are shown in Table 1. The fermentation broth of *B. megaterium* was uniformly sprayed on the surface of the commercial material at a rate of 100 ml/kg, and then, the sprayed feed was placed in a cool place for 1 h, after which it was used as the experimental group feed. The control group was fed the unaltered commercial feed.

### 2.2. Feeding Experiment

Songpu mirror carp (*Cyprinus specularis* Songpu) (body weight 77.75 ± 3.15g) were obtained from the Hulan Experimental Station of the Heilongjiang River Fisheries Research Institute in Heilongjiang Province, China (45.97°N, 126.63°E). The fish were acclimatized to the laboratory conditions for 14 days and adapted to the experimental control feed prior to the experiment. Then, 90 healthy Songpu mirror carp were selected and randomly distributed into two groups (control group and experimental group). Each treatment was performed in triplicate, and each replicate had 15 fish. The control group was fed commercial feed. The treatment group was fed commercial feed coated with *B. megaterium*. The daily feeding amount was 3% of the body weight of the Songpu mirror carp, three times per day at 08 : 00, 12 : 00, and 19 : 00. The entire experimental period was 56 days. During the experimental period, the laboratory water was replaced by 1/3 of the water volume per week. The water quality was measured (using YSI professional plus, Ohio State, USA) daily during the experimental period, the water temperature ranged from 18 to 25°C, and an air compressor was used to add oxygen 24 h per day. The fish were weighed both at the start and at the end of the feeding trial.

### 2.3. Sample Collection

Before the feeding experiment, ten fish were randomly taken from the temporary culture tank, and their initial body weights were measured. Then, the intestinal tract was collected for the measurement of the initial intestinal microflora (initial group). At the end of the 56-day feeding trial, approximately 24 h after the last feeding, all fish were anesthetized with MS-222 diluted in the water at a concentration of 75 mg/l. These fish were counted and weighed to determine the weight gain rate (WGR), specific growth rate (SGR), and feed conversion rate (FCR, Table 2) [21].

After obtaining the final weight of all fish, five fish from each tank were randomly selected and placed in an ice plate for rapid dissection. Tissue samples, including the foregut, midgut, and hindgut, were collected with aseptic scissors, washed with aseptic physiological saline, weighed, and then prepared into a homogenate with aseptic physiological saline (1 : 4). All samples were immediately stored at −80°C in a freezer for digestive enzyme determination. Another five fish were also sampled randomly from each tank. The surfaces of the fish were disinfected with 75% alcohol before the fish were taken into a bioclean room. After further disinfection, the abdominal cavity was opened, the exterior of the intestine was wiped with 75% alcohol and washed four times with sterile water, and then the intestinal tract was collected for evaluation of the final intestinal microflora.

### 2.4. Digestive Enzyme Determination

Before measuring the immunity indexes, the pooled foregut, midgut, and hindgut were manually homogenized in a glass homogenizer with 0.86% NaCl (*w*/*v*) to obtain a 10% homogenate. After centrifugation (4000 rpm, 10 min) at 4°C, the supernatant, consisting primarily of crude enzyme liquid, was obtained. The activities of amylase, protease, and lipase in the foregut, midgut, and hindgut were analyzed spectrophotometrically using diagnostic reagent kits (Nanjing Jiancheng Bioengineering Institute, China).

### 2.5. 16S rRNA Gene Amplification and Illumina Sequencing

Microbial DNA was extracted from the intestinal samples using the E.Z.N.A.® Soil DNA Kit (Omega Bio-Tek, Norcross, GA, USA) according to the manufacturer's protocols. The final DNA concentration and purification were determined with a NanoDrop 2000 UV-vis spectrophotometer (Thermo Scientific, Wilmington, USA), and the DNA quality was determined by 1% agarose gel electrophoresis. The V4-V5 hypervariable regions of the bacterial 16S rRNA genes were amplified with primers 515F (5′-GTGCCAGCMGCCGCGG-3′) and 907R (5′-CCGTCAATTCMTTTRAGTTT-3′) by using a thermocycler PCR system (GeneAmp 9700, ABI, USA). The PCRs were conducted using the following program: 3 min of denaturation at 95°C, 27 cycles of 30 s at 95°C, 30 s for annealing at 55°C, and 45 s for elongation at 72°C, and a final extension at 72°C for 10 min. The resulting PCR products were extracted from a 2% agarose gel and further purified using an AxyPrep DNA gel extraction kit (Axygen Biosciences, Union City, CA, USA) and quantified using QuantiFluor™-ST (Promega, USA) according to the manufacturer's protocol. Purified amplicons were pooled in equimolar amounts and paired-end sequenced (2 × 300) on an Illumina MiSeq platform (Illumina, San Diego, USA) according to the standard protocols by Majorbio Bio-Pharm Technology Co., Ltd. (Shanghai, China) [22].

### 2.6. Statistical Analysis

Operational taxonomic units (OTUs) were clustered with a 97% similarity cutoff using UPARSE (version 7.1, http://drive5.com/uparse/) with a novel “greedy” algorithm that simultaneously performs chimera filtering and OTU clustering. The taxonomy of each 16S rRNA gene sequence was analyzed by the RDP Classifier algorithm (http://rdp.cme.msu.edu/) against the SILVA 16S rRNA database using a confidence threshold of 70%. The Chao and ACE estimator indexes were selected to identify the community richness, and the Shannon and Simpson indexes were used to identify the community diversity [23]. All of these indexes in our samples were calculated with QIIME (version 1.7.0) and prepared for display with R software (version 2.15.3). The statistical analyses were performed with the statistical software package SPSS 20.0 (SPSS, Chicago, IL, USA). The data are expressed as the mean ± SD of three replicates. The data were subjected to one-way ANOVA, and when differences were found, the means were ranked using Duncan's multiple comparison test. Differences were considered significant at *P* < 0.05.

## 3. Results

### 3.1. Effect of the *B. megaterium*-Coated Diet on Fish Growth

As shown in Table 2, the weight gain rate and specific growth rate of the treatment group (71.03%, 0.42% day^−1^) were significantly increased compared with those of the control group (52.75%, 0.33% day^−1^, *P* < 0.05), and the feed conversion ratio of the treatment group (1.28) was significantly lower than that of the control group (2.23, *P* < 0.05). No mortality was observed during the 56 days of the feeding trial.

### 3.2. Effect of the *B. megaterium*-Coated Diet on Digestive Enzyme Activity

The effects of the *B. megaterium*-coated diet on digestive enzyme activity are shown in Table 3. The foregut amylase activity in the treatment group was significantly higher than that in the control group (*P* < 0.05). The midgut and hindgut amylase activities in the treatment group were significantly higher than those in the control group, but there were no significant differences (*P* > 0.05). The foregut, midgut, and hindgut protease activities of the treatment group were higher than those of the control group, but there were no significant differences (*P* > 0.05). The foregut lipase activity in the treatment group was significantly higher than that in the control group (*P* < 0.05). The midgut and hindgut lipase activities in the treatment group were basically the same as those in the control group, and there were no significant differences (*P* > 0.05).

### 3.3. Effect of the *B. megaterium*-Coated Diet on Intestinal Microbial Diversity and Richness

The intestinal microbial diversity of the Songpu mirror carp was determined by high-throughput sequencing. The average number of OTUs detected from the initial group sample was 63. After 56 days, the average number of OTUs detected in the control and treatment groups was 114 and 196, respectively. The coverage index of all samples was above 0.97, indicating that there was a high detection rate. As shown in Table 4, the Chao and ACE indexes, which reflect the richness of the intestinal microflora of Songpu mirror carp, were significantly higher in the treatment group after 56 days than in the control and initial groups (*P* < 0.05). The Shannon index of intestinal community diversity in the treatment group was significantly higher than that in the control group (*P* < 0.05). The Simpson index of intestinal community diversity in the treatment group was significantly lower than that in the control group (*P* < 0.05). There were no significant differences for the above four indexes among the control and initial groups (*P* > 0.05).

### 3.4. Effect of the *B. megaterium*-Coated Diet on the Composition and Changes in the Main Microbiota in the Intestine of the Songpu Mirror Carp

**Figure 1:**
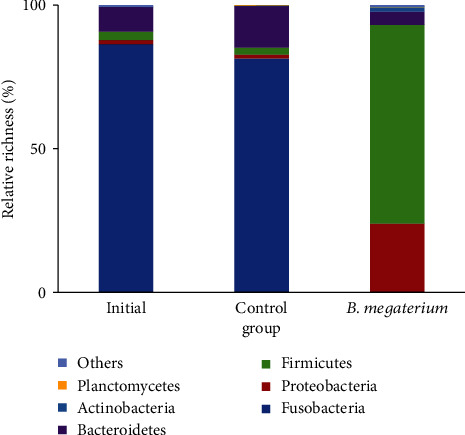
Intestinal microbial compositions at the phylum level in Songpu mirror carp fed a diet coated with *B. megaterium* for 56 days.

An intestinal microbial richness of more than 1% at the phylum level was taken as the main microflora for the statistics presented in Figure 1. The dominant phylum in the intestine of the initial group was predominantly Fusobacteria (86.36%) and Bacteroidetes (8.60%) before the experiment began. After the 56-day feeding trial, the number of the main intestinal microflora increased in each group. In the control group, the dominant phyla were Fusobacteria (86.36%), Bacteroidetes (14.4%), Firmicutes (2.42%), and Proteobacteria (1.37%). In the treatment group, the dominant phyla were Firmicutes (69.15%), Proteobacteria (23.8%), Bacteroidetes (4.65%), and Actinobacteria (1.43%). An intestinal microbial richness of more than 0.5% at the genus level was taken as the main microflora for the statistical calculations, shown in Table 5. There were only 5 genera of the main intestinal microorganisms in the initial group. After the 56-day feeding trial, the main intestinal microorganisms increased to 11 genera in the treatment group. The most abundant microorganism in the intestine of Songpu mirror carp in the initial group was *Cetobacterium*. After the 56-day feeding trial, the most abundant microorganism in the control group was still *Cetobacterium*. In the control group, the 11 dominant genera were *Bacillus*, *Lactococcus*, *Pseudomonas*, *Stenotrophomonas*, *Psychrobacter*, *Brochothrix*, *Myroides*, *Arthrobacter*, *Flavobacterium*, *Comamonadaceae*_unclassified, and *Yersinia*. The Songpu mirror carp fed a diet coated with *B. megaterium* displayed increased proportions of intestinal *Bacillus* and *Lactococcus* at the genus level, which were both significantly higher than those of the control group (*P* < 0.05).

## 4. Discussion

Probiotics such as *Bacilli* have been widely used in aquaculture. Some studies have shown that the addition of various strains of *Bacillus* spp. to larva feed has achieved good results and has a good promoting effect on the growth of fish larvae [24, 25]. The addition of bacteria (strain CA2) as a food supplement to xenic larval cultures of the oyster *Crassostrea gigas* consistently enhanced the growth of the larvae during different seasons of the year [26]. Manipulation of microbiota using probiotics has been reported as a worthy practice for aquaculture to control or inhibit pathogenic bacteria and to improve growth performance and the activity of digestive enzymes [27]. In comparison to the untreated control group, the final weight and weight gain were significantly greater in shrimp fed a mixture of two probiotic strain diets [28]. In this study, it was found that a *B. megaterium*-coated diet increased the weight gain rate and specific growth rate and reduced the feed conversion ratio, indicating that *B. megaterium*-coated diets can promote the growth of Songpu mirror carp. Similar results were found in catfish *Clarias* sp., where the addition of *B. megaterium* PTB 1.4 to their feed significantly improved their growth rate [29]. Similar results were also found in *Penaeus monodon*, for which higher FCR and SGR values were obtained after the addition of *Bacillus cereus* [30].

Probiotic bacteria are capable of producing digestive enzymes that help fish use feed nutrients and digest them [31]. The addition of *B. megaterium* to plant protein meals can promote intestinal morphology development and increase digestive enzyme activity [8]. The study of probiotics for the common carp *Cyprinus carpio* based on growth performance and digestive enzyme activities showed that the mean digestive enzyme activities of all probiotic treatment groups were significantly different from that of the control [27]. In the present study, a *B. megaterium*-coated diet had a positive and important effect on digestive enzyme activities, especially those of amylase and protease in the foregut, midgut, and hindgut. Similar results were also found in tilapia, which showed improvement in food digestion and growth after *Bacillus* NP5 was added to their feed [32]. A higher level of enzyme activity obtained with diets containing probiotics improved the digestion of protein, starch, fat, and cellulose, which might, in turn, explain the better growth observed with the probiotic-supplemented diets [24]. Digestive enzymes help fish break down and digest nutrients in feed, making it easier for the fish to absorb the nutrients in the feed [29].

Advances in high-throughput sequencing have enabled an extensive catalog of metagenomic samples, providing insight into the diversity of microbial species from a wide variety of sources, including the ocean, soil, and human body. These studies use both 16S rRNA gene sequencing to determine phylogenetic relationships and more comprehensive shotgun sequencing to predict the detailed species and gene composition [33]. The richness index and diversity index are important indexes to detect the diversity and complexity of microorganisms in samples [2, 34]. In this study, with the growth of Songpu mirror carp, the intestinal microbial diversity and richness were increased, and the Chao and ACE indexes, which reflect the richness of the intestinal microflora of Songpu mirror carp, were significantly higher in the treatment group than in the control group. In addition, the Shannon index of intestinal community diversity in the treatment group was significantly higher than that in the control group. Similarly, the results also showed that the intestinal microflora structure was changed when juvenile blunt snout bream were fed diets supplemented with different levels of *Bacillus subtilis* [3, 35]. The addition of probiotics to feed can also change the number and structure of the original microflora in the intestinal tract of *Litopenaeus vannamei* and promote the complex interactions between the microbial communities in the intestinal tract of *Litopenaeus vannamei* [36].

In this study, the Songpu mirror carp fed a diet coated with *B. megaterium* displayed increased proportions of intestinal *Bacillus* and *Lactococcus* at the genus level, which were both significantly higher than those of the control group. The results also indicated that the composition and proportions of the main microbiota in the intestine of Songpu mirror carp can be changed with *B. megaterium*-coated diets. In conclusion, the use of *B. megaterium*-coated diets can significantly enhance fish growth and reduce the feed conversion ratio, improve the activities of digestive enzymes, and enrich the beneficial genus composition of the main intestinal microflora.

## Figures and Tables

**Table 1:** Product composition analysis guaranteed value of commercial feed (%).

Item	Contents
Nutrient levels	
Crude protein	≥32.0
Crude lipid	≥3.00
Crude fiber	≤10.0
Crude ash	≤16.0
Total phosphorus	≥0.50
Lysine	≥2.00
Moisture	≤12.0

**Table 2:** Growth of Songpu mirror carp (*Cyprinus specularis* Songpu) fed diets coated with *B. megaterium* for 56 days (*n* = 3).

Treatment	Control	*B. megaterium*
Initial body weight (g)	77.75 ± 3.15	77.75 ± 3.15
Final body weight (g)	118.78 ± 5.52	129.44 ± 10.78
WGR (%)	52.75 ± 1.04^a^	71.03 ± 4.11^b^
SGR (% day^−1^)	0.33 ± 0.01^a^	0.42 ± 0.02^b^
FCR	2.23 ± 0.17^a^	1.28 ± 0.10^b^

Note: in the same row, values with different small letter superscripts mean significant difference (*P* < 0.05), while those with the same or no letter superscripts mean no significant difference (*P* > 0.05). Weightgainrate(WGR, %) = 100 × (finalbodyweight − initialbodyweight)/initialbodyweight. Specificgrowthrate(SGR, %day^−1^) = 100 × (lnfinalbodyweight − lninitialweight)/days. Feedconversionratio(FCR) = dryfeedconsumed(g)/(finalbodyweight − initialbodyweight).

**Table 3:** Activities of digestive enzymes in the intestine (foregut, midgut, and hindgut) of Songpu mirror carp fed diets coated with *B. megaterium* (U/g protein).

Treatment	Foregut		Midgut		Hindgut	
	Control	*B. megaterium*	Control	*B. megaterium*	Control	*B. megaterium*
Amylase	13.86 ± 6.24^a^	64.64 ± 7.93^b^	67.46 ± 17.73	39.57 ± 17.80	55.37 ± 4.63	56.03 ± 5.69
Protease	2.02 ± 0.04	2.02 ± 0.24	2.26 ± 0.05	2.38 ± 0.14	1.52 ± 0.04^a^	1.87 ± 0.19^b^
Lipase	520.13 ± 90.28^a^	973.29 ± 50.12^b^	925.27 ± 110.81	941.74 ± 170.45	706.31 ± 46.15	671.14 ± 150.31

Note: in the same column, values with different small letter superscripts mean significant difference (*P* < 0.05), while those with the same or no letter superscripts mean no significant difference (*P* > 0.05).

**Table 4:** Effects of *B. megaterium*-coated diets on abundance and diversity of the intestinal microflora of Songpu mirror carp.

Groups	Enrichment index		Diversity index	
	Chao	ACE	Simpson	Shannon
Initial	123.52 ± 9.79^a^	120.63 ± 11.78^a^	0.42 ± 0.13^a^	0.87 ± 0.04^a^
Control	137.66 ± 20.65^a^	140.93 ± 19.10^a^	0.59 ± 0.01^a^	0.99 ± 0.01^a^
*B. megaterium*	204.49 ± 8.81^b^	205.24 ± 7.90^b^	0.11 ± 0.01^b^	2.66 ± 0.01^b^

Note: in the same column, values with different small letter superscripts mean significant difference (*P* < 0.05), while those with the same or no letter superscripts mean no significant difference (*P* > 0.05).

**Table 5:** Percentages of the main genus of the intestinal microflora in Songpu mirror carp fed a diet coated with *B. megaterium* for 56 days.

Genus	Treatments		
	Initial	Control	*B. megaterium*
*Cetobacterium*	46.33 ± 11.25	75.75 ± 15.28	—
*Lactococcus*	—	—	27.46 ± 11.32
*Bacillus*	—	—	34.83 ± 9.48
*Enterobacteriaceae_*unclassified	—	—	—
*Bacteroides*	9.46 ± 6.45	11.89 ± 8.46	—
*Comamonadaceae_*unclassified		—	0.78 ± 0.43
*Yersinia*	0.32 ± 0.21	—	0.63 ± 0.53
*Pseudomonas*	—	—	11.33 ± 8.73
*Pseudoxanthomonas*	—	—	—
*Fusobacteriales_*unclassified	3.84 ± 2.98	5.66 ± 4.12	—
*Stenotrophomonas*		—	5.66 ± 4.23
*Psychrobacter*	—	—	3.94 ± 2.84
*Leucobacter*	—	—	—
*Brochothrix*	—	—	3.03 ± 2.13
*Myroides*		—	2.97 ± 1.96
*Barnesiella*	1.12 ± 1.01	2.46 ± 1.32	—
*Rhodobacter*		—	—
*Arenimonas*		—	—
*Clostridium*		—	—
*Arthrobacter*		—	1.36 ± 0.96
*Flavobacterium*		—	1.07 ± 0.83
Others	2.94 ± 1.46	2.85 ± 1.56	7.31 ± 4.23

Notes: — means that the percentage of the genus accounting for the total intestinal microflora is less than 0.5%.

## Data Availability

All data was provided in the article, and there are no more data to be uploaded.
